# Use of analgesics in acute stroke patients with inability to self-report pain: a retrospective cohort study

**DOI:** 10.1186/s12883-020-1606-x

**Published:** 2020-01-14

**Authors:** J. Schuster, C. Hoyer, A. Ebert, A. Alonso

**Affiliations:** Department of Neurology, Medical Faculty of Mannheim, University of Heidelberg, Theodor Kutzer Ufer 1-3, 68167 Mannheim, Germany

**Keywords:** Stroke, Pain, Inability to communicate, Consciousness disorder, Analgesia

## Abstract

**Background:**

Pain is a common and burdensome complication in patients with acute stroke. We assessed the impact of impaired communication in stroke patients on pain assessment and treatment.

**Methods:**

We included 909 (507 male, mean age 71.8 years) patients admitted to our stroke unit from 01/2015 to 12/2015 in the analysis. Patients were assigned to four groups: able to communicate (AC), not able to communicate prior to index stroke (P-NAC), due to focal symptoms of index stroke (S-NAC), due to a reduced level of consciousness (C-NAC). Pain prevalence, documentation of pain and use of analgesics were evaluated. C-NAC patients were excluded from analyses regarding analgesic treatment due to relevant differences in patient characteristics.

**Results:**

746 patients (82.1%) were classified as AC, 25 (2.8%) as P-NAC, 90 (9.9%) as S-NAC and 48 (5.3%) as C-NAC. Pain was documented on the Numeric Rating Scale and in form of free text by nurses and physicians. Nurses documented pain more frequently than physicians (*p* < 0.001). Pain prevalence was 47.0% (n.s. between groups). The use of analgesic medication increased from 48.7% in the AC group, to 76.0% in the P-NAC group, and 77.8% in the S-NAC group (*p* < 0.001). Opioid use was significantly more frequent in NAC patients (p < 0.001). The response to the treatment was poorly documented with significantly lowest rates in S-NAC patients (*p* < 0.001).

**Conclusions:**

Our study suggests that post-stroke pain in patients with inability to communicate is not attended enough, not systematically assessed and therefore not sufficiently treated.

## Background

Stroke is one of the leading causes of death and disability worldwide [1]. Recent decades have seen a decline in case fatality, which appears to be mainly attributable to progress and improvement of acute treatment and care after stroke as well as of primary and secondary prevention of vascular risk factors [2]. However, medical complications are still a frequent and relevant problem in stroke patients due to their association with increased post-stroke mortality and disability [3].

With prevalence estimates ranging from 10 to close to 50%, post-stroke pain (PSP) is common [4–8]. The most frequently occurring PSP syndromes are headache [6, 9], musculoskeletal pain, shoulder pain [6, 8, 10, 11], complex regional pain syndrome [12] and central post-stroke pain [13]. Not only do patients suffer from the pain itself, the occurrence of post-stroke pain also correlates with fatigue, depression, sleep disorders, increased mortality [7] and suicidality [10] as well as slower rehabilitation progress [14, 15]. Pain after stroke is an under-recognized [16] and under-assessed phenomenon, and it is usually assumed that communication difficulties in stroke patients are a major contributing reason [17]. In addition, a reliable and validated tool for PSP assessment is lacking, and common methods of pain assessment are not straightforwardly applicable in stroke patients [18]. As a consequence of these insufficiencies in recognition and assessment, PSP is frequently not sufficiently attended to [19].

Impairment or loss of the ability to communicate, which may be due to hearing, vision, speech, language or cognitive problems, is a common problem in stroke patients. At least one communication-related impairment was found in 88% of patients in an acute stroke unit [20]. Disturbance of verbal communication in particular can be ascribed to various reasons. To begin with, aphasia and dysarthria may occur as focal deficits subsequent to a stroke event, affecting up to 25 and 40% of all stroke patients, respectively [21–24]. Reduced level of consciousness may also be a direct consequence of strokes particularly of the thalamus or the brainstem [25]. Previous studies found the prevalence of impaired consciousness to range between 5 and 40%, depending on the timing of assessment after stroke [26–30]. Delirium which affects relevant proportions of stroke patients [31], may also impact on language use and hence substantially interfere with successful communication [32]. Pre-existing structural brain pathology, e. g. post-traumatic, may also contribute to impair patients’ ability to communicate, as can pre-existing cognitive impairment, for example in the context of a neurodegenerative disorder like dementia. Finally, the presence of a language barrier is an important non-medical reason for communication problems and has been demonstrated to negatively impact on patient safety [33, 34].

There is a paucity of research investigating both the assessment and treatment of pain in patients post-stroke who are not able to communicate. To the best of our knowledge, only one dedicated study exists [35], which, however, focused on the epidemiology of this phenomenon rather than looking at ensuing therapeutic consequences. Hence, the current study aims at answering the question how impaired communication in stroke patients impacts on pain assessment and treatment.

## Methods

The patient cohort for this retrospective cohort study was extracted from our prospectively collected stroke database. All patients admitted to our comprehensive stroke unit (SU) between January 2015 and December 2015 were screened (*n* = 1055). Patients with a final diagnosis of ischemic stroke, intracerebral hemorrhage (ICH) or transient ischemic attack (TIA) were included. Clinical data including baseline characteristics, stroke syndrome, clinical scores (National Institute of Health Stroke Scale, NIHSS; Glasgow Coma Scale, GCS; modified Rankin Scale, mRS), stroke treatment, and length of hospital stay as well as technical investigations were extracted from the stroke database. Information on pain assessment and medication was collected by analysing the patient charts. Pain assessment followed the standard of care on our stroke unit, comprising estimation of pain on the numeric rating scale (NRS) every 8 h. In addition, nurses and physicians documented the kind of pain the patients were suffering from, and whether it responded to analgesics or not (freetext). In patients unable to communicate, behavioral and physiologic parameters indicative of the presence of pain were utilized in pain assessment. These were: sounds (moaning), movement including guarding movements for certain areas of the body, facial expression, muscular tension, restlessness as well as vital signs suggestive of stress (rise in blood pressure and/or heart rate, sweating). Pain was divided into chronic pain, defined as pain syndrome with onset present at least three months prior to the index stroke [36, 37], and acute pain, defined as a pain syndrome with onset after the index stroke. Pain syndromes were classified as headache, musculoskeletal, pain associated with chronic diseases, other pain syndromes and multiple pain syndromes. Analgesics were classified as opioids, non-opioids and co-analgesics. Indication, dosage and change in dosage over time were recorded. Frequency of documentation concerning pain intensity after the initiation of analgesic treatment was recorded.

Patients were classified as not able to communicate prior to index stroke (P-NAC) if lack of communicability (both verbal and non-verbal) together with an explaining comorbidity (dementia, prior stroke, prior brain trauma, CNS malignoma) was documented in the patient charts. Classification as not able to communicate as a consequence of focal symptoms of index stroke (S-NAC) required documented lack of communicability (both verbal and non-verbal) together with an explaining stroke symptom (severe aphasia, defined as at least 2 points in item 9/best language on NIHSS for more than 50% of hospital stay; severe dysarthria, defined as 2 points in item 10/dysarthria on NIHSS for more than 50% of hospital stay) in patients with unimpaired consciousness. Patients with a Glasgow Coma Scale score of < 12 during more than 50% of the hospital stay were classified as not able to communicate due to a reduced level of consciousness (C-NAC). All remaining patients were subsumed under the category “able to communicate” (AC). C-NAC patients were excluded from analyses regarding analgesic treatment due to relevant differences in patient characteristics.

Statistical analysis was performed using the Statistical Package for the Social Sciences (SPSS), version 24 (IBM, USA). Data are reported as frequency (n) and percentage (%), mean and standard deviation SD) or median and interquartile range (IQR) as applicable.

Group comparisons were calculated by a Chi^2^-Test or in case of small cell sizes Fisher’s Exact Test for nominally scaled variables. In case of overall significant differences between the groups, two-by-two comparisons were done through 4-Fields- Chi^2^-Tests or Fisher’s Exact Tests. For metric variables we performed a one-way ANOVA, followed by post-hoc independent samples t-tests**.** A *p* value < 0.05 was considered to indicate statistical significance. Bonferroni correction was omitted due to the explorative character of the study.

## Results

### Characteristics of the study population – demographics, index stroke

We included a total of 909 patients in our analysis, 507 (55.8%) male. Demographic data are presented in Table 1.

**Table 1:** Baseline characteristics

	AC	P-NAC	S-NAC	C-NAC	*p*-value
*Number of patients*, *n* (%)	746 (82.1)	25 (2.8)	90 (9.9)	48 (5.3)	
Demographics					
age, years, mean (SD)	70.46 (15.55)	79.6 (10.94)	77.4 (12.56)	78.27(13.59)	< 0.001^abc^
sex, male, *n* (%)	427 (57.2)	14 (56.0)	50 (55.6)	16 (33.3)	0.015^cf^
living circumstances prior to index stroke					< 0.001^abcde^
independent at home, *n* (%)	669 (90.0)	5 (20.0)	53 (60.2)	29 (60.4)	
family care, *n* (%)	51 (6.9)	9 (36.0)	19 (21.6)	9 (18.8)	
institutional care, *n* (%)	23 (3.1)	11 (44.0)	16 (18.2)	10 (20.8)	
premorbid mRS score >/= 3, *n* (%)	92 (12.4)	20 (80.0)	26 (29.5)	18 (38.3)	< 0.001^abcde^
Vascular risk factors					
atrial fibrillation	168 (22.5)	16 (64.0)	50 (55.6)	24 (50.0)	< 0.001^abc^
hypertension, *n* (%)	612 (82.0)	21 (84.0)	83 (92.2)	44 (91.7)	0.03^b^
diabetes mellitus, *n* (%)	205 (27.5)	11 (44.0)	26 (28.9)	11 (22.9)	n.s.
dyslipidemia, *n* (%)	285 (38.2)	9 (36.0)	29 (32.2)	15 (31.3)	n.s.
coronary heart disease, *n* (%)	166 (22.3)	9 (36.0)	34 (37.8)	11 (22.9)	0.006^b^
prior myocardial infarction, *n* (%)	87 (11.7)	1 (4.0)	17 (18.9)	7 (14.6)	n.s.
prior stroke, *n* (%)	175 (23.5)	11 (44.0)	27 (30.0)	18 (37.5)	0.013^ac^
current or past smoker, *n* (%)	197 (26.4)	4 (16.0)	10 (11.1)	3 (6.3)	< 0.001^bc^
Comorbidities					
Dementia, *n* (%)	58 (7.8)	21 (84.0)	20 (22.2)	7 (14.6)	< 0.001^abde^
Depression, *n* (%)	63 (8.4)	6 (24.0)	9 (10.0)	6 (12.5)	n.s.
Cancer, *n* (%)	33 (4.4)	1 (4.0)	9 (10.0)	0 (0.0)	0.049^bf^
Index stroke					
Ischemia, *n* (%)	621 (83.2)	23 (92.0)	77 (85.6)	38 (79.2)	
Intracerebral hemorrhage, *n* (%)	50 (6.7)	1 (4.0)	13 (14.4)	10 (20.8)	
TIA, *n* (%)	75 (10.1)	1 (4.0)	0 (0.0)	0 (0.0)	
NIHSS at admission, median (IQR)	3 (5)	10 (12)	17.5 (12)	23 (8)	< 0.001^abcef^
severe aphasia, *n* (%)	67 (9.0)	10 (40.0)	67 (74.4)	28 (59.6)	< 0.001^abcd^
severe dysarthria, *n* (%)	51 (6.8)	9 (36.0)	39 (43.3)	24 (50.0)	< 0.001^abc^
GCS < 12 at admission, *n* (%)	2 (0.3)	1 (4.0)	7 (7.8)	18 (37.5)	< 0.001^bcef^
Length of hospital stay, days, mean (SD)	11.2 (6.6)	10.6 (6.9)	13.3 (8.3)	9.2 (7.7)	< 0.001^bcf^
In-hospital death, *n* (%)	1 (0.1)	5 (20.0)	10 (11.1)	37 (77.1)	< 0.001^abcdef^

a, significant group difference AC vs P-NAC

b, significant group difference AC vs S-NAC

c, significant group difference AC vs C-NAC

d, significant group difference P-NAC vs S-NAC

e, significant group difference P-NAC vs C-NAC

f, significant group difference S-NAC vs C-NAC

Of all patients, 746 (82.1%) were classified as AC, 25 (2.8%) as P-NAC, 90 (9.9%) as S-NAC and 48 (5.3%) as C-NAC. Patients categorized as AC were significantly younger than patients of the other groups (*p* < 0.001). In their medical history, both P-NAC and C-NAC patients had significantly more often suffered a prior stroke compared to patients of the AC group (*p* = 0.031). Likewise, the prevalence of dementia was highest in the P-NAC group (*p* < 0.001).

Overall, 759/909 (83.5%) of stroke events were ischemic strokes. In 51.2% the territory of the medial cerebral artery was affected, in 27.9% the territory of the anterior cerebral artery, in 29.5% the territory of the posterior cerebral artery (with affection of the thalamus in 14.6%), in 8.9% the brainstem and in 7.8% the cerebellum. In addition, there were 74/909 cases (8.1%) of ICH. Seventy-six patients (8.4%) had a final diagnosis of TIA. The C-NAC group had the highest proportion of ICH patients (10/48, 20.8%) while the AC group had the highest proportion of TIA patients (75/746, 10.1%). Accordingly, NIHSS at admission varied significantly between the groups with increasing values from AC patients (median NIHSS 3) over P-NAC and S-NAC patients (median NIHSS 10 and 18, respectively) to C-NAC patients (median NIHSS 23). Stroke severity was further reflected in in-hospital-mortality rates, with 0.1% in AC patients compared to 11.1% in S-NAC patients, 20.0% in P-NAC patients and 77.1% in C-NAC patients. Severe aphasia and severe dysarthria were significantly more frequent in NAC patients. The highest proportion of severely aphasic patients was found in the S-NAC group (74.4%) whereas the C-NAC group included the highest rate of severely dysarthric patients (50.0%).

### Pain assessment and analgesic treatment (C-NAC patients excluded)

Documentation rates of pain, comprising NRS and/or freetext documentation, were significantly higher in nurses’ records (any documentation of pain in 855 of 861 patients, 99.3%) than in physicians’ records (any documentation of pain in 231 of 861 patients, 26.8%; *p* < 0.001). Pain documentation by nurses did not differ significantly between the groups whereas physicians documented pain significantly least often in the AC group (p < 0.001). NRS was implemented in 848 patients (98.5%) at any time point, least frequently in S-NAC patients (Table 2).

**Table 2:** Assessment of pain and use of analgesics

	AC	P-NAC	S-NAC	p-value
Number of patients, *n* (%)	746 (86.6)	25 (2.9)	90 (10.5)	
Assessment of pain				
Any, *n* (%), documented by nurses	741 (99.5)	25 (100.0)	89 (98.9)	n.s.
Any, *n* (%), documented by physicians	161 (21.6)	13 (52.0)	57 (63.3)	< 0.001^ab^
NRS, *n* (%)	738 (99.1)	25 (100.0)	85 (94.4)	< 0.012^b^
NRS, frequency, *n* per day (SD)	2.44 (1.10)	2.02 (1.33)	1.68 (0.96)	n.s.
Pain prevalence				
Total, *n* (%)	353 (47.3)	11 (44.0)	38 (42.2)	n.s.
Headache, *n* (%)	188 (25.2)	4 (16.0)	10 (11.1)	0.008^b^
Musculoskeletal pain, *n* (%)	82 (11.0)	1 (4.0)	13 (14.4)	n.s.
Chronic pain syndrome, *n* (%)	36 (4.8)	0 (0.0)	1 (1.1)	n.s.
Not specified, *n* (%)	199 (26.7)	7 (28.0)	26 (28.9)	n.s.
Use of analgesics				
Any analgesic, *n* (%)	363 (48.7)	19 (76.0)	70 (77.8)	< 0.001^ab^
Opioid, *n* (%)	54 (7.2)	5 (20.0)	16 (17.8)	0.001^ab^
Non-Opioid, *n* (%)	278 (37.3)	11 (44.0)	56 (62.2)	< 0.001^b^
Co-analgesic, *n* (%)	165 (22.1)	13 (52.0)	21 (23.3)	0.002^ac^
Indication				
Acute or chronic pain, *n* (%)	245 (32.8)	3 (12.0)	22 (24.4)	0.029^a^
Prophylactic treatment, *n* (%)	3 (0.4)	5 (20.0)	13 (14.4)	< 0.001^ab^
Not documented, *n* (%)	64 (8.6)	5 (20.0)	18 (20.0)	0.001^b^
Other, *n* (%)	148 (19.8)	15 (60.0)	43 (47.8)	0.000^ab^
Documented response to treatment, *n/n* (%)	120/245 (49.0)	1/3 (33.3)	2/22 (9.1)	< 0.001^b^

a, significant group difference AC vs P-NAC

b, significant group difference AC vs S-NAC

c, significant group difference P-NAC vs S-NAC

402/861 patients (46.7%) suffered from pain during the hospitalisation after stroke. Headache was the most frequent pain syndrome (202 patients, 23.5%), followed by musculoskeletal pain (96 patients, 11.1%). Patients with ICH suffered significantly more frequently from pain (61.5%, *p* < 0.001) than patients with ischemic stroke or TIA (47.4, and 27.6%, respectively). The prevalence of headache also differed significantly between the aetiologies (22.8% in ischemic stroke patients, 36.9% in ICH patients, 18.4% in TIA patients, *p* = 0.020).

Regarding the use of analgesia, 48.7% of AC patients received any analgesic medication. The proportions of patients receiving medication increased in the P-NAC and S-NAC groups, with 76.0 and 77.8%, respectively. The P-NAC and S-NAC group, in whom 20.0 and 17.8% of patients, respectively, were treated with opioids, differed significantly from the AC group (*p* < 0.001). S-NAC patients also received more non-opioids than the patients of the AC group (56/90, 62.2% vs 278/746, 37.3%; p < 0.001). P-NAC patients were significantly more often treated with a co-analgesic than all other patients (*p* = 0.002).

The proportion of patients who received an analgesic because of manifest acute or chronic pain did not differ significantly between the groups. Moreover, the AC group received analgesics less often because of other effects like lowering fever (*p* = 0.000).

Documentation rate concerning the clinical course of pain after the initiation of analgesic treatment in patients with manifest pain was overall low. Respective documentation was found in 49% of AC patients, but only in one of the three patients in the P-NAC group and in 9.1% of S-NAC patients (*p* < 0.001).

## Discussion

To our knowledge, this is the first study to analyse both the assessment and treatment of pain in post-stroke patients who are not able to communicate.

Our main findings are that pain after stroke is a common phenomenon but inconsistently assessed in patients with limited ability to self-report pain. As a consequence, there are relevant differences with regard to the extent and type of analgesic treatment in these patients in comparison to others who are able to communicate normally.

The American Society for Pain Management Nursing states that all persons with pain deserve prompt recognition and treatment. In patients who are unable to self-report pain, assessment strategies must be adapted [38]. In the proposed Hierarchy of pain Assessment Techniques for patients with limited verbal and/or cognitive skills, self-report of pain remains heads, followed by a search for potential causes of pain, observation of patient behaviour, proxy reporting and attempt of an analgesic trial [39]. In our stroke unit, the NRS is used by nursing staff to assess pain by self-report. The NRS is a commonly utilized method for the systematic assessment of pain, and a modified version was found to be the most discriminative self-report scale in comparison to other assessment scales in critically ill patients [40]. However, as their name implies, self-report scales critically rely on patients‘understanding of the - usually verbally transmitted – concept to assign a number or graphic item to the level of pain experienced as well as to either say that number or point to the respective item. Patients with aphasia, dementia or reduced level of consciousness, however, are frequently unable to do just that, so that the usefulness and value of self-report scales is drastically limited [41]. Although there are pain scales specifically designed for patients with limited or missing ability to self-report pain, e. g. the Faces Pain Scale [42, 43], the Behavioral Pain Scale [44], the Pain Assessment Checklist for Seniors with Limited Ability to Communicate [45] or the Critical-Care Pain Observation Tool [46], their use is still problematic in many clinical situations, for example in patients with brain injury [47].

While nurses and physicians have been demonstrated to exhibit similar degrees of pain assessment accuracy [48], nurses document pain more frequently than physicians [49]. Our data corroborate this finding, which is presumably due to nurses’ more frequent and differently-natured types of contact with patients as well as having to keep record of the NRS. Physicians’ documentation rates, on the contrary, were overall low. Interestingly, documentation rates were lowest in AC patients and highest in S-NAC patients, possibly reflecting a certain awareness of physicians for the consequences of impaired communication. Still, physicians did not record pain-related information, i. e. absence or presence of any kind of pain, in over 75% of cases in our cohort.

Even with those limitations regarding pain evaluation, we found 47% of all patients were experiencing pain at any time point in the acute phase after stroke. This is in line with other studies in stroke populations [4–7]. In comparison, patients with dementia admitted to general hospitals – sharing several co-morbidities with our study population-, the prevalence of pain was lower when relying on self-report of the patients only [50]. However, with implementation of an observational tool, the pain prevalence on movement increased up to 57% [50]. This finding stresses the importance not only of implementing an appropriate assessment tool but also of the evaluation of potentially painful procedures.

In our cohort, pain was more frequent in patients suffering from ICH than from ischemic stroke or TIA. The prevalence of specific kinds of pain is not directly comparable to other studies, as those assessed either only chronic [4–7] or acute pain [6]. Of note, the prevalence of headache during hospitalization was most frequent in haemorrhagic stroke patients. This is in line with previous findings showing that headache at the time of stroke onset is significantly more frequent in ICH patients [9].

We only analysed the number of patients whose records had at least one mentioning or description of pain in it. This parameter does not reflect the fluctuating course of pain syndromes with the result that recurrent episodes of pain may not have been recognized. A good indicator for this issue is the fact that many patients in the NAC groups had the ability to communicate pain at some point during their hospitalization and their doing so may suggest that they were in pain while being unable to communicate as well. An American study showed similar results: 47.6% of patients who were able to self-report pain on the day of hospital dismissal, but not on admission, had a pain score above zero [35]. Pain in the NAC groups was often inferred to be present by nursing staff when they observed patients moaning while being repositioned. Even though behavioural observation is a component principle of certain pain scales, patients with traumatic and non-traumatic brain-injury may show different behavioural responses to pain [51].

Retrospectively, we are not able to tell how many patients suffered from pain directly attributable to stroke. Since we analysed patient records from the hospitalisation for acute stroke, post-stroke pain occurring with greater latency, e.g. like most types of central post-stroke pain [52], went unnoticed.

We noted relevant differences in analgesia use between groups of communication impaired and unimpaired stroke patients: the NAC groups received significantly more often at least one analgesic than the AC group. However, the indication for analgesia use was only to a small extent the treatment of pain. More often analgesics were prescribed with a different primary intention such as antipyresis. This may be in part explicable through the fact that medical complications such as post-stroke infections occur more frequently in severe strokes [53], which predominantly affected patients of the NAC groups. The majority of C-NAC patients underwent prophylactic treatment of pain – often with opioids – as for many of those patients not regaining consciousness during the hospitalization, the therapy concept was changed from curative to palliative [54, 55]. We therefore excluded these patients from analyses regarding analgesic treatment. In addition, in the NAC groups, the indication of analgesics was not documented in up to 20%, whereas in the AC group, an indication was missing in less than 10%, which presumably is a direct consequence and reflection of the difficulties in assessing the localisation and intensity of pain in NAC patients. Data regarding the use of different types of pain medication in patients with reduced or missing ability to self-report pain are inconsistent: cognitively impaired patients were found to receive less analgesics – opioids in particular – in some studies [56, 57] but not others [58]. In patients suffering from dementia, physicians are frequently reluctant to prescribe opioids due to the higher rates of complications and side-effects [59]. However, in our study, significantly more NAC patients received opioids than patients in the AC group. Again, the shift to a palliative care setting, which has different aims and focus from usual stroke care and concerned C-NAC patients in particular, may be one explanation for this finding. Nevertheless, our findings indicate that pharmacological pain management in NAC patients did mostly not follow the World Health Organization analgesic ladder for pain management. Although prescription rates of non-opioids in the S-NAC group exceeded those in the AC group, this excess is presumably attributable to the common use of paracetamol and metamizole to lower fever in the severely ill NAC patients as reflected by the higher NIHSS in NAC patients.

Higher rates of analgesia in patients not able to communicate do not necessarily equal sufficient or adequate treatment of pain. One finding strongly suggestive of suboptimal pain management is the lack of documented response to treatment, with not even half of AC patients’ response to analgesia documented, and lowest rates in the S-NAC group. Similarly, a Canadian study investigating pain assessment of intubated patients also found a re-assessment rate of only 40% after an intervention intended to manage pain [49].

Major limitation of our study is the retrospective character, allowing for several potential sources of bias. Nonetheless, the retrospective nature may best reflect the results from daily routine whereas prospective studies may yield optimistic documentation rates due to an attention bias. A further limitation is the arbitrary assignment to a NAC group if lack of communicability was comprehensible over more than 50% of the hospital stay. We are well aware that construct validity may be impaired by utilizing behavioral and physiologic parameters indicative of the presence of pain in a non-formalized way in patients unable to communicate and thus relying on nurses’ and physicians’ Gestalt perceptional impression. In addition, we did not formally assess treatment response to analgesia, which would certainly have been informative since this represents the major patient-related outcome of any intervention regarding the management of pain. The inclusion of a control group would potentially increase validity, which, however, was not possible on the basis of our retrospective body of data. Finally, as our study was conceived with an explorative character in order to generate hypotheses, we omitted a Bonferroni correction in our statistical analyses. Further prospective studies will thus be needed to corroborate our data.

## Conclusions

Our study suggests that pain in patients with limitations or inability to communicate is not attended to enough, not systematically assessed and therefore not sufficiently treated during hospitalisation for acute stroke. Strategies to improve recognition and treatment of pain in patients with inability to self-repost should comprise a bundle of measures: With different types of reasons for impaired communication in stroke patients, a particular challenge lies in the appropriate, systematic and consistent use of different types of pain scales adjusted to patients’ communicative skills as a crucial first step in improving management of pain; Best practice recommendations regarding pharmacological and non-pharmacological pain management should be established; Recognition and management of pain should be considered as an interprofessional team approach; Specialised pain nurse advocates may promote permanent and active involvement in the complex issue of pain management in stroke patients [17].

## Data Availability

The datasets used and/or analysed during the current study are available from the corresponding author on reasonable request.

## Abbreviations

AC: Able to communicate
C-NAC: Not able to communicate due to a reduced level of consciousness
GCS: Glasgow Coma Scale
ICH: Intracerebral haemorrhage
IQR: Interquartile range
mRS: Modified Rankin Scale
NIHSS: National Institute of Health Stroke Scale
NRS: Numeric rating scale
P-NAC: not able to communicate prior to index stroke
PSP: Post-stroke pain
SD: Standard deviation
S-NAC: Not able to communicate due to focal symptoms of index stroke
SU: Stroke unit
TIA: Transient ischemic attack
